# Profiles of progesterone and bovine interferon-τ in repeat breeding and non-repeat breeding Aceh cows

**DOI:** 10.14202/vetworld.2021.230-236

**Published:** 2021-01-26

**Authors:** Husnurrizal Husnurrizal, Tongku Nizwan Siregar, Gholib Gholib, Budianto Panjaitan, Teuku Armansyah, Sri Wahyuni

**Affiliations:** 1 Laboratory of Reproduction, Faculty of Veterinary Medicine, Universitas Syiah Kuala, Banda Aceh 23111, Indonesia; 2 Laboratory of Physiology, Faculty of Veterinary Medicine, Universitas Syiah Kuala, Banda Aceh 23111, Indonesia; 3 Laboratory of Clinic, Faculty of Veterinary Medicine, Universitas Syiah Kuala, Banda Aceh 23111, Indonesia; 4 Laboratory of Pharmacology, Faculty of Veterinary Medicine, Universitas Syiah Kuala, Banda Aceh 23111, Indonesia; 5 Laboratory of Anatomy, Faculty of Veterinary Medicine, Universitas Syiah Kuala, Banda Aceh 23111, Indonesia

**Keywords:** Aceh cows, bovine interferon-τ, repeat breeding

## Abstract

**Aim:**

This study aimed at determining the profiles of progesterone and bovine interferon-τ (bIFN-τ) and the correlation between the two in repeat breeding (RB) Aceh cattle and non-RB Aceh cattle.

**Materials and Methods:**

The study was performed on five RB and five non-RB Aceh cows. These cows were subjected to estrous synchronization using the prostaglandin F2 alpha hormone, which was followed by artificial insemination (AI). Serum samples were collected on days 5, 6, 7, 15, 16, and 17 after AI to measure the concentration of progesterone at the beginning and end of the luteal phase and from days 14 to 18 after AI to measure the concentration of bIFN-τ. The concentrations of progesterone and bIFN-τ were determined using enzyme-linked immunosorbent assay. Pregnancy examinations were performed by ultrasonography on days 25, 35, 45, and 55 after AI. Data for progesterone and bIFN-τ concentrations were analyzed using the Mann–Whitney and t-tests, and the correlation between progesterone and bIFN-τ was analyzed using the Spearman correlation test.

**Results:**

The average concentration of progesterone in RB Aceh cows tended to be lower than non-RB Aceh cows, but it was not significantly different (p>0.05). Similar results also found in the concentration of bIFN-τ which RB Aceh cows tended to have lower bIFN-τ concentrations compared to non-RB Aceh cows, but it was also not significantly different (p>0.05). Moreover, the concentrations of progesterone and bIFN-τ in RB and non-RB Aceh cows did not show a significant correlation (p>0.05). These results of the ultrasonography showed that non-RB Aceh cows were pregnant from day 25 to day 55 after AI, whereas RB Aceh cows were not pregnant and had early embryonic death.

**Conclusion:**

The concentrations of progesterone and bIFN-τ in non-RB Aceh cows tended to be higher than those in RB Aceh cows, although, it was not significantly different. Non-RB Aceh cows were able to maintain pregnancy until day 55, whereas RB Aceh cows were diagnosed with early embryonic death before day 25 after AI.

## Introduction

One of the reproductive disorders in Aceh cattle is repeat breeding (RB), which has an incidence rate of 58.3% [1,2]. This disorder occurs in cows that have a normal estrous cycle but experiences pregnancy failures after mating more than 3 times and shows no clear clinical symptoms of disease or abnormalities in their reproductive organs [3]. Factors that cause RB in cattle can be genetic [4], infections of the vagina, cervix, and uterus, anatomical abnormalities of the reproductive organs [5], uterine and ovarian dysfunction, and obstruction of the oviduct [6], hormonal disorders [7], and oocyte defects that can cause pregnancy failure, early embryonic death, and endocrine disorders [6]. Several studies have shown phenomenon of reproductive disorders in cows that can result in the high incidence rate of RB. The incidence of RB is also associated with hormonal imbalance, such as the low concentration of hormone progesterone [8]. One of the causes of RB in Aceh cattle is hormonal imbalance, particularly in the progesterone levels during the peak luteal phase [2]. The peak concentration of progesterone in Aceh cattle was reported to be only 1.54 ng/mL on day 13 of the estrous cycle [9]; however, according to Naik *et al*. [10], the peak concentration reached 10.66 ng/mL on day 15 of the cycle. Furthermore, the low conception rate and high embryo mortality rate in Aceh cattle are assumed to be due to low progesterone concentrations at the peak of the luteal phase. The low concentration of progesterone is one of the suspected causes of early embryonic death and RB in Aceh cattle [11].

Apart from low progesterone levels, early embryonic death is also associated with interference between the release of signals (communication) of the blastocysts and the main endometrium or maternal recognition of pregnancy (MRP) [12-14]. One of the MRP signals in ruminants is interferon-tau (IFN-τ) [15,16], also known as bovine IFN-τ (bIFN-τ) in cattle, which is produced from day 14 of pregnancy [17,18], when the blastocyst starts the elongation process [16], until day 21 [19,20]. Failure of the IFN-τ release mechanism[21] and hormonal disturbances [22] at the onset of pregnancy can cause early embryonic death [23,24]. This is consistent with the report of Kose *et al*. [25], a decrease in the concentration of bIFN-τ in cows with early embryonic death. In addition, the production of IFN-τ from embryos in RB cattle is low [26]. Production of bIFN-τ is closely related to the uterine immune regulatory system during early gestation [27]. It is assumed that bIFN-τ is a key factor in sustaining the early development of embryo until implantation during early pregnancy. This phenomenon is thought to occur in Aceh cows that experience RB [28]. To maintain pregnancy, a synergistic working mechanism between progesterone and bIFN-τ is needed [29]. The concentration of progesterone in early pregnancy is closely related to the elongation process of the cows’ conceptus (embryo) and the increase in bIFN-τ production [15,30-33]. There are a correlation and interaction between the expression of progesterone and bIFN-τ at the onset of the gestation period [34,35].

Although there are reports that IFN-τ cannot be assayed directly from the blood circulation [36], according to Antoniazzi *et al*. [37], it can be detected on day 15 from the uterine veins in sheep. Because the IFN-τ action occurs in paracrine and endocrine manner, it is suspected that IFN-τ can be detected from the peripheral circulation. Our preliminary study has proven that the level of IFN-τ in Aceh cows could be detected in serum from the 14^th^ day of pregnancy.

Therefore, it is assumed that the IFN-τ profile can be used as an indicator of RB occurrence in Aceh cows. In the present study, we evaluated the profiles of progesterone and bIFN-τ in the serum and determined the correlation between the two in RB and non-RB Aceh cows to obtain a better understanding of RB.

## Materials and Methods

### Ethical approval

All the experiments on animals performed in this study were approved by the Animal Ethics Committee of the Faculty of Veterinary Medicine of Universitas Syiah Kuala, Indonesia (Ref No. 65/KEPH/X/2020).

### Study period and location

The study was conducted from May to October 2019. Sample collection, artificial insemination (AI), and ultrasonography examination were carried out at Balai Pembibitan Ternak Unggul dan Hijauan Pakan ternak (BPTU-HPT), Indrapuri, Aceh, Indonesia. Progesterone and bovine IFN-τ measurements were performed at Laboratory of Physiology, Faculty of Veterinary Medicine, Universitas Syiah Kuala.

### Animals

The study was conducted on 10 Aceh cattle, aged 3-8 years, with regular estrous cycles and body condition scores above 3. Each cow had given birth at least once. These cows included five non-RB Aceh cattle, namely, cows that had normal estrous cycles with a service rate per conception <3, and five RB Aceh cattle, namely, cows that had mated or were artificially inseminated more than 3 times but failed to get pregnant.

### Estrous synchronization and AI

The estrous levels of all the cows were synchronized using 5 mL of prostaglandin F2 alpha (PGF2α) hormone (Lutalyse™, Pharmacia and Upjohn Company, Pfizer Inc.) administered intramuscularly, with a double injection (twice at a gap of 11 days) [2]. After the injection of PGF2α, estrous levels were measured twice daily at 08.00 and 16.00 for 30 min each. The estrous was observed visually. The cows would show signs of primary and secondary estrous, such as standing heat, riding other cattle, restlessness, red and swollen vulva, discharge of cervical mucus, and decreased appetite. AI was carried out using frozen semen from Aceh bulls, 12-18 h after estrous appeared.

### Blood serum sample collection

Blood sample collection was carried out in two stages. The first stage was to check progesterone levels and was carried out on days 5, 6, and 7 after AI, and the second stage was used to check the bIFN-τ levels and was carried out on days 14, 15, 16, 17, and 18 after AI. The collected serum was placed in a microtube and stored in a freezer at −20°C before analysis.

### Measurement of the progesterone concentration

Progesterone concentration was determined using ELISA according to the procedure described in the ELISA progesterone kit manual (DRG, GmbH Instrument, Germany). The procedure was started by dispensing 25 μL of the standard solution, sample, and control into the wells of microplates, which were then incubated for 30 min at room temperature (20-23°C). Next, 200 μL of the enzyme conjugate progesterone was added to each well of the microplates and stirred for 10 s for complete mixing. This solution was then incubated for 60 min at room temperature (20-23°C). Next, the solution was shaken quickly so that the contents of the well were removed, and the well was washed 3 times by adding 400 μL of wash solution to each well. The microplate was then tapped on an absorbent paper quickly to remove any remaining droplets. Next, 200 μL of substrate solution was added to each well and incubated for 15 min at room temperature. The enzymatic reaction was stopped by adding 100 μL of stop solution to each well. The absorbance values were read on a microtiter plate reader at a wavelength of 450±10 nm within 10 min.

### Measurement of the bIFN-τ concentration

The concentration of bIFN-τ was determined by ELISA as recommended in the manual of the bIFN-τ ELISA kit (Cusabio Technology LLC AII, USA). The measurement was started by preparing the bIFN-τ ELISA kit, reagent, standard solution, and serum samples. First, 100 μL of standard solution and sample was dispensed into each well of a microplate and incubated for 2 h at 37°C. The solution was then removed from the well (the plate was not washed). Next, 100 μL of biotin antibody was added to each well and incubated for 1 h at 37°C. All contents of the wells were subsequently aspirated. This process was repeated twice, and the microplates were washed 3 times with 200 µL washing buffer. Thereafter, 100 µL of horseradish peroxidase avidin was added to each well, incubated for 1 h at 37°C, and aspirated. The microplates were washed 5 times. After the wash, 90 µL TMB substrate was added to the wells and incubated for 15-30 min at 37°C away from light. Finally, the enzymatic reaction was stopped by adding 50 µL stop solution to all the wells, and absorbance was read at a wavelength of 450 nm within 5 min.

### Examination of pregnancy

Examination of pregnancy was performed using ultrasonography (Shenzhen Mindray Bio-Medical Electronics Co., Ltd.) on days 25, 35, 45, and 55 after AI [38]. The cows were considered pregnant on day 25 after AI based on the presence of anechoic fluid with visualization of the embryo and heart rate in one of the uterine cornua. Embryonic death was diagnosed on day 35 based on non-embryonic visibility, absence of positive signs of pregnancy, or signs of embryonic degeneration.

### Statistical analysis

Data on the concentrations of progesterone and bIFN-τ were analyzed using the Mann–Whitney U-test and t-test. To determine the correlation between the concentrations of progesterone and bIFN-τ, the Spearman correlation test was used. Statistical analysis was performed using SPSS 24.0 (IBM Corp. NY, USA).

## Results

**Figure-1:**
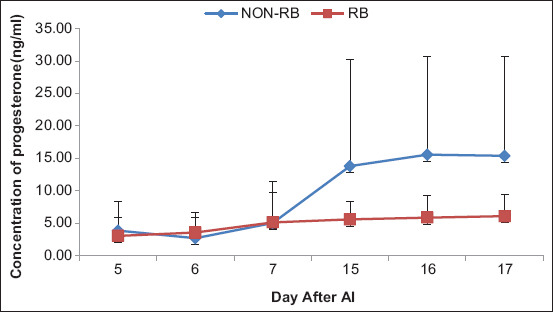
The progesterone concentration in repeat breeding (RB) Aceh cows and non-RB Aceh cows from day 14 to day 18 after artificial insemination (p>0.05).

The concentrations of progesterone tended to be lower in RB Aceh cows compared to non-RB cows. However, results of statistical analysis showed that there was no significant difference between the progesterone concentrations in RB and non-RB cows (p>0.05) (Figure-1). Although there were no significant differences during the first examination period (days 5-7), differences in progesterone concentrations between the two groups began to appear starting day 15 after AI.

**Figure-2:**
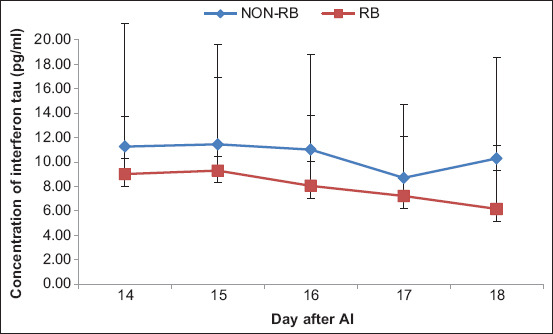
The bovine interferon-τ (bIFN-τ) concentration in repeat breeding (RB) Aceh cows and non-RB Aceh cows from day 14 to day 18 after artificial insemination. The concentration of bIFN-τ in the two groups of Aceh cows showed no significant difference (p>0.05).

**Figure-3:**
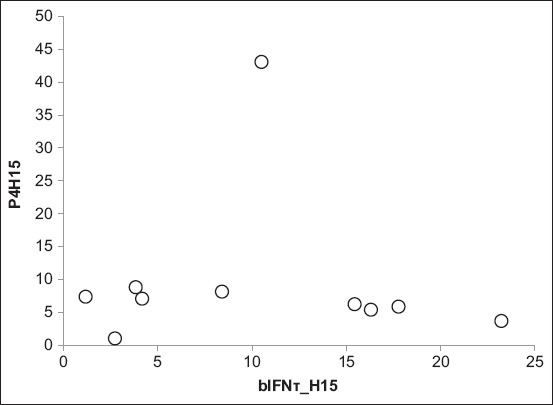
The correlation between progesterone and bovine interferon-τ in in repeat breeding (RB) Aceh cows and non-RB Aceh cows

The concentrations of bIFN-τ in RB Aceh cows also tended to have lower compared to non-RB cows. However, it was not significantly different (p>0.05) (Figure-2). Moreover, the concentrations of progesterone in the RB and non-RB Aceh cattle had no significant correlation with those of bIFN-τ from day 15 to day 17 after AI (p>0.05) (Figure-3).

## Discussion

There was no significant difference in the concentrations of progesterone between RB and non-RB Aceh cows from day 5 to day 7 after AI. This result differs from that obtained for Ongole cows that experienced RB in the early luteal phase, in which case, the concentration of progesterone was lower than that in fertile cows [39]. This discrepancy might be due to the differences in examination patterns. In this study, the progesterone concentration was determined in only one cycle, whereas Widayati *et al*. [39] determined the concentrations in two consecutive cycles with the aim of reducing the variations among individuals. However, one of the causes of RB is the low level of progesterone. Progesterone supplementation has been shown to increase the pregnancy rate in RB cows.

The concentration of progesterone in non-RB Aceh cows on day 15 after AI was significantly increased compared to that in RB cows. The concentration of progesterone in non-RB Aceh cattle on day 15 after AI was higher than that in RB cattle. The progesterone concentration on day 16 and day 17 after AI was also similar to that on days 15, 16, and 17.

Cows that fail to conceive typically show a decrease in progesterone concentrations after day 15 post-AI [40]. Furthermore, the concentration of progesterone in cows that were not pregnant increased until day 15 and decreased due to the lysis of the corpus luteum, but pregnant cows had higher concentrations of progesterone than cows that were not pregnant on the same day [41]. A high concentration of progesterone affects the optimal uterine environment, which is important for the growth and development of the embryo [42]. In addition, a high level of progesterone stimulates embryonic growth during the critical phase of pregnancy [43].

**Figure-4:**
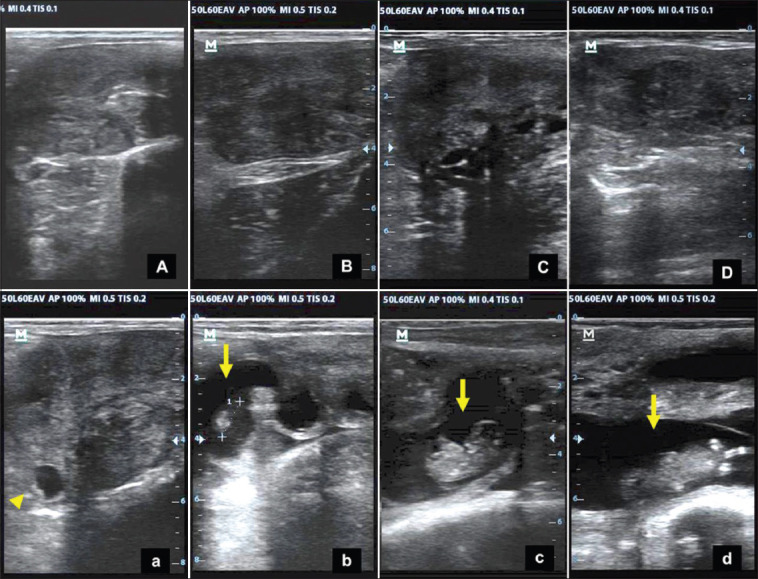
A uterine overview of an repeat breeding (RB) and non-RB Aceh cows; The uterus of an RB Aceh cow on days 25, 35, 45, and 55 after artificial insemination (AI) (A, B, D, and D); and a non-RB Aceh cow on days 25, 35, 45, and 55 after AI (a, b, c, and d). The arrows indicate the presence of fetus; the arrow heads show the conceptual vesicles with fetus only as dots on the white on the conceptual wall of the vesicles.

The results obtained in this study show that the progesterone concentration in non-RB cows was higher than that in RB cows, which could be the reason that the non-RB cows were able to maintain pregnancy. This is supported by the results of ultrasonography from day 25 to day 55 after AI (Figure-4). Increasing the concentration of progesterone after AI has a good effect on embryo development and strengthens the MRP system in the parent, such as the expression of bIFN-τ.

To maintain successful pregnancy in cows, a synergistic working mechanism between progesterone and bIFN-τ is needed [29]. High progesterone concentrations are closely related to the production of IFN-τ, which plays a direct role in increasing the success of pregnancy [30,33,34]. In both the groups of Aceh cows (RB and non-RB), the presence of bIFN-τ in the serum was detected from day 14 to day 18 after AI. This is consistent with the results of Romero *et al*. [44] who reported the detection of IFN-τ in cattle on day 15 and day 16. Basavaraja *et al*. [17] detected IFN-τ until day 18 of pregnancy, whereas Yaginuma *et al*. [26] reported an increase in the activity of bIFN-τ (mRNA expression of ISG15) and MX2 protein in the serum from day 14 to day 21 of pregnancy. This increase was not observed in cows that were not pregnant.

The concentration of bIFN-τ in the non-RB Aceh cows was higher than that in the RB Aceh cows, although statistically there were insignificant differences from day 15 to day 18 after AI. This finding is consistent with that of Serrano-Pérez *et al*. [34] who reported a higher expression of the IFN-τ-stimulated gene (ISG15) in pregnant cows than in non-pregnant cows.

The concentration of bIFN-τ in the non-RB Aceh cows on day 14 after AI was higher than that in the RB Aceh cows. This is in accordance with the results of a previous study showing that the concentration of bIFN-τ (mRNA expression from ISG15) in pregnant RB cows was significantly higher than that in non-pregnant RB cows [45]. An increase in the concentration of bIFN-τ was also detected on day 14 after AI[46] and in pregnant cows compared to that in non-pregnant cows [28].

The concentration of bIFN-τ in the non-RB Aceh cows on day 15 after AI was higher than that in RB cows. An increase in the average concentration of bIFN-τ was observed in both the groups of Aceh cows on day 15 relative to that on day 14. The increase in the concentration of bIFN-τ indicated an increase in embryo development in the non-RB Aceh cows. Furthermore, the concentration of bIFN-τ on day 16 after AI was still significantly higher in non-RB Aceh cows than in RB cows. The average concentration of bIFN-τ on day 16 was lower than that on day 14 and day 15 after AI. Thus, the concentration of bIFN-τ on day 15 was the peak concentration in Aceh cows. Different findings were reported by Bai *et al*. [46] who showed the maximum production of IFN-τ on day 16 of pregnancy, just before implantation or the attachment of the conceptus to the uterine epithelium. The peak production of IFN-τ in goats occurs between days 15 and 17 of pregnancy. The expression of IFN-τ decreases rapidly during the implantation process.

The concentration of bIFN-τ in the non-RB Aceh cows on day 17 and day 18 after AI was also higher than that in RB cows. The results show a decrease in the average concentration of bIFN-τ in the two Aceh cow groups compared to that on day 16. However, the concentration of bIFN-τ on day 18 in the non-RB Aceh cows increased. Based on the concentration of bIFN-τ, the two groups of Aceh cows were equally capable of getting pregnant. However, the RB Aceh cows were suspected of being unable to maintain pregnancy and of experiencing early embryonic death. This is indicated by the low concentrations of progesterone and bIFN-τ and the results of examinations from day 25 to day 55 after AI.

The possibility of the RB Aceh cows experiencing early embryonic death is supported by the low concentrations of progesterone on days 15-17 after AI, although bIFN-τ was still produced by the conceptus (embryo) until day 18 after AI. Despite this, ultrasonography on day 25 showed negative results (not pregnant). The low concentration of bIFN-τ in the RB Aceh cows compared to that in the non-RB Aceh cows indicates a difference in the quality of conceptus. Rizos *et al*. [47] reported that IFN-τ is only produced by the trophectoderm conceptus, and there is a positive correlation between the length of the conceptus and the level of IFN-τ secretion. Aceh cattle that have lower IFN-τ concentrations have poor conceptus quality, which can lead to early embryonic death.

The production of IFN-τ from RB cattle embryos was low. Thus, IFN-τ should be added at the beginning of pregnancy to increase the conception rate in RB cattle [26]. During early pregnancy, the production of bIFN-τ is closely related to the uterine immune regulatory system [27,48]. This can be a key factor in sustaining the embryonic development in the early period until implantation during early pregnancy [28]. In addition, other factors originating from the embryo at the beginning of pregnancy might also be involved in immunological interactions between the embryo and immune cells that interact with the local system in the mother’s reproductive tract, building a tolerance that results in the mother’s immune system accepting the embryos during the early stages of pregnancy [48].

Progesterone and bIFN-τ are very important components in the early stages of pregnancy. In ruminants, high progesterone concentrations are closely related to the conceptus elongation process in cattle [30] and to the increased production of IFN-τ [15,31-35]. The concentrations of progesterone in RB and non-RB Aceh cows had no significant correlation with the concentration of bIFN-τ on days 15-17 after AI. This finding is different from that of other studies wherein a strong positive correlation between progesterone, conceptus elongation, and bIFN-τ production was observed on day 14 after embryonic transfer [47]. The weak correlation in this study was likely due to the time difference in analyzing the correlation. In this study, the correlation analysis was carried out on day 15 for both progesterone and bIFN-τ secretion. The effect of progesterone on day 7 will affect the expression of bIFN-τ on day 15 after *in vitro* fertilization [49].

## Conclusion

We conclude that the concentrations of progesterone and bIFN-τ in non-RB Aceh cows were higher than in RB Aceh cows and there was a non-significant correlation between the concentrations of bIFN-τ and progesterone in RB and non-RB Aceh cattle (p>0.05). Non-RB Aceh cattle were still able to maintain pregnancy until day 55, whereas RB Aceh cows experienced early embryonic death before day 25 after AI.

## Authors’ Contributions

HH, TNS, SW design the study, collection of samples, data analysis, and drafted the manuscript. GG and BP reviewed the manuscript and did hormone analysis. TA supervised the field and laboratory works. All the authors read and approved the final manuscript.
